# Supplementary material for: Response-based outcome predictions and confidence regulate feedback processing and learning
Source: eLife. 2021 Apr 30;10:e62825. doi: 10.7554/eLife.62825 (PMC8121545; doi:10.7554/eLife.62825)
Supplement: Supplementary file 1. [file elife-62825-supp1.docx]

**Table S1.** *Follow-up on Prediction and Performance Precision effects on Confidence*

|  | **Confidence** | | | | |
| --- | --- | --- | --- | --- | --- |
| *Predictors* | *Estimates* | *SE* | *CI* | *t* | *p* |
| (Intercept) | 0.26 | 0.04 | 0.18 – 0.33 | 6.36 | **2.055e-10** |
| Block 2-1 | 0.03 | 0.01 | 0.01 – 0.06 | 2.98 | **2.859e-03** |
| Block 3-2 | 0.01 | 0.01 | -0.01 – 0.03 | 0.77 | 4.424e-01 |
| Block 4-3 | 0.03 | 0.01 | 0.01 – 0.06 | 3.03 | **2.424e-03** |
| Block 5-4 | 0.01 | 0.01 | -0.01 – 0.03 | 1.05 | 2.942e-01 |
| Block [1] : SPE | -0.43 | 0.06 | -0.55 – -0.30 | -6.74 | **1.535e-11** |
| Block [2] : SPE | -0.47 | 0.07 | -0.61 – -0.34 | -6.76 | **1.350e-11** |
| Block [3] : SPE | -0.35 | 0.07 | -0.48 – -0.21 | -5.05 | **4.431e-07** |
| Block [4] : SPE | -0.46 | 0.07 | -0.60 – -0.33 | -6.75 | **1.431e-11** |
| Block [5] : SPE | -0.63 | 0.07 | -0.76 – -0.49 | -9.13 | **6.882e-20** |
| Block [1] : Error Magnitude | 0.03 | 0.06 | -0.09 – 0.16 | 0.53 | 5.964e-01 |
| Block [2] : Error Magnitude | 0.15 | 0.07 | 0.01 – 0.29 | 2.14 | **3.219e-02** |
| Block [3] : Error Magnitude | 0.11 | 0.07 | -0.03 – 0.24 | 1.50 | 1.341e-01 |
| Block [4] : Error Magnitude | 0.27 | 0.07 | 0.13 – 0.41 | 3.69 | **2.286e-04** |
| Block [5] : Error Magnitude | 0.26 | 0.07 | 0.11 – 0.40 | 3.51 | **4.561e-04** |
| **Random Effects** |  |  | **Model Parameters** |  |  |
| Residuals | 0.12 |  | N | 40 | |
| Intercept | 0.06 |  | Observations | 9996 | |
| SPE | 0.03 |  | log-Likelihood | -3786.661 | |
| Error Magnitude | 0.06 |  | Deviance | 7573.321 | |

*Formula: Confidence ~ Block/( Error Magnitude+ SPE) + (Error Magnitude+SPE|participant);*

*Note: “:” indicates interactions*
