# Supplementary material for: Response-based outcome predictions and confidence regulate feedback processing and learning
Source: eLife. 2021 Apr 30;10:e62825. doi: 10.7554/eLife.62825 (PMC8121545; doi:10.7554/eLife.62825)
Supplement: Supplementary file 2. [file elife-62825-supp2.docx]

**Table S2.** *Control analysis for Confidence Calibration effect on Learning*

|  | **Log Error Magnitude** | | | | |
| --- | --- | --- | --- | --- | --- |
| *Predictors* | *Estimates* | *SE* | *CI* | *t* | *p* |
| (Intercept) | 5.05 | 0.06 | 4.93 – 5.18 | 80.64 | **0.000e+00** |
| Confidence Calibration | 0.31 | 0.56 | -0.78 – 1.40 | 0.55 | 5.796e-01 |
| Trial (linear) | -0.46 | 0.07 | -0.59 – -0.33 | -6.83 | **8.486e-12** |
| Trial (quadratic) | 0.12 | 0.02 | 0.07 – 0.16 | 5.03 | **4.784e-07** |
| Response Variance | 0.54 | 0.05 | 0.43 – 0.64 | 9.96 | **2.391e-23** |
| Trial (linear) : Confidence Calibration | -0.66 | 0.31 | -1.27 – -0.05 | -2.12 | **3.393e-02** |
| **Random Effects** |  |  | **Model Parameters** |  |  |
| Residuals | 1.17 |  | N | 40 | |
| Intercept | 0.11 |  | Observations | 9996 | |
| Trial (linear) | 0.03 |  | log-Likelihood | -15057.557 | |
|  |  |  | Deviance | 30115.114 | |

*Formula: log Error Magnitude ~ Confidence Calibration*Trial (linear)+ Trial (quadratic)+ Response Variance+ (Trial(linear) |participant); Note: “:” indicates interactions*
