# Supplementary material for: Response-based outcome predictions and confidence regulate feedback processing and learning
Source: eLife. 2021 Apr 30;10:e62825. doi: 10.7554/eLife.62825 (PMC8121545; doi:10.7554/eLife.62825)
Supplement: Supplementary file 3. [file elife-62825-supp3.docx]

**Table S3.** *Follow-up on* *Block and Confidence effects on Relative Error Signals*

|  | **Absolute RPE vs EM** | | | | **RPE vs EM** | | | | **SPE vs EM** | | | |
| --- | --- | --- | --- | --- | --- | --- | --- | --- | --- | --- | --- | --- |
| *Predictors* | *Estimates* | *SE* | *t* | *p* | *Estimates* | *SE* | *t* | *p* | *Estimates* | *SE* | *t* | *p* |
| (Intercept) | -40.99 | 3.78 | -10.84 | **2.167e-27** | -115.03 | 8.42 | -13.66 | **1.855e-42** | 1.90 | 3.39 | 0.56 | 5.760e-01 |
| Block | 19.44 | 3.98 | 4.89 | **1.002e-06** | -0.58 | 5.38 | -0.11 | 9.137e-01 | 18.49 | 4.29 | 4.31 | **1.647e-05** |
| Confidence | -25.84 | 9.43 | -2.74 | **6.124e-03** | -7.14 | 19.33 | -0.37 | 7.120e-01 | -54.05 | 9.74 | -5.55 | **2.883e-08** |
| Block : Confidence | -5.29 | 4.94 | -1.07 | 2.844e-01 | 38.44 | 5.28 | 7.28 | **3.334e-13** | -8.24 | 5.61 | -1.47 | 1.418e-01 |
| **Random Effects** | | | | | | | | | | | | |
| Residuals | 16863.59 | | | | 18142.16 | | | | 22090.63 | | | |
| Intercept | 443.78 | | | | 2671.03 | | | | 296.41 | | | |
| Confidence | 2873.45 | | | | 14032.18 | | | | 2952.20 | | | |
| Block | 426.60 | | | | 927.54 | | | | 469.39 | | | |
| N | 40 | | | | 40 | | | | 40 | | | |
| Observations | 9996 | | | | 9996 | | | | 9996 | | | |
| Deviance | 125867.367 | | | | 126731.891 | | | | 128536.108 | | | |
| log-Likelihood | -62933.683 | | | | -63365.945 | | | | -64268.054 | | | |

*Formula: DV ~ Block* Confidence + (Block + Confidence |participant); DVs are Absolute RPE vs Error Magnitude, RPE vs Error Magnitude*

*and SPE vs Error Magnitude; Note: “:” indicates interactions*
