# Supplementary material for: Response-based outcome predictions and confidence regulate feedback processing and learning
Source: eLife. 2021 Apr 30;10:e62825. doi: 10.7554/eLife.62825 (PMC8121545; doi:10.7554/eLife.62825)
Supplement: Supplementary file 4. [file elife-62825-supp4.docx]

**Table S4.** *Follow-up on Confidence by Block Interaction on RPE benefit over Error Magnitude*

|  | **RPE vs Error Magnitude** | | | | |
| --- | --- | --- | --- | --- | --- |
| *Predictors* | *Estimates* | *SE* | *CI* | *t* | *p* |
| (Intercept) | -114.67 | 8.41 | -131.16 – -98.18 | -13.63 | **<0.001** |
| Block2-1 | 12.08 | 5.46 | 1.37 – 22.79 | 2.21 | **0.027** |
| Block3-2 | -1.93 | 5.50 | -12.71 – 8.84 | -0.35 | 0.725 |
| Block4-3 | -3.21 | 5.58 | -14.15 – 7.72 | -0.58 | 0.565 |
| Block5-4 | -6.58 | 5.70 | -17.75 – 4.59 | -1.16 | 0.248 |
| Block [1] : Confidence | -46.80 | 20.49 | -86.95 – -6.64 | -2.28 | **0.022** |
| Block [2] : Confidence | -20.33 | 20.25 | -60.01 – 19.35 | -1.00 | 0.315 |
| Block [3] : Confidence | -19.37 | 20.14 | -58.85 – 20.10 | -0.96 | 0.336 |
| Block [4] : Confidence | 15.10 | 20.21 | -24.52 – 54.72 | 0.75 | 0.455 |
| Block [5] : Confidence | 35.03 | 20.46 | -5.07 – 75.14 | 1.71 | 0.087 |
| **Random Effects** | | | | | |
| Residual | 18110.80 | | | | |
| Intercept | 2663.50 | | | | |
| Confidence | 13872.45 | | | | |
| Block | 929.76 | | | | |
| N | 40 | | | | |
| Observations | 9996 | | | | |
| Deviance | 126714.387 | | | | |
| log-Likelihood | -63357.194 | | | | |

*Formula: RPE vs Error Magnitude ~ Block/Confidence +(Confidence|participant);*

*Note: “:” indicates interactions*
