# Supplementary material for: Response-based outcome predictions and confidence regulate feedback processing and learning
Source: eLife. 2021 Apr 30;10:e62825. doi: 10.7554/eLife.62825 (PMC8121545; doi:10.7554/eLife.62825)
Supplement: Supplementary file 5. [file elife-62825-supp5.docx]

**Table S5.** *Block and Confidence effects on Error Signals*

|  | **Error Magnitude** | | | | | | **RPE** | | | | | **SPE** | | | | |
| --- | --- | --- | --- | --- | --- | --- | --- | --- | --- | --- | --- | --- | --- | --- | --- | --- |
| *Predictors* | *Estimates* | | *SE* | *CI* | *t* | *p* | *Estimates* | *SE* | *CI* | *t* | *p* | *Estimates* | *SE* | *CI* | *t* | *p* |
| (Intercept) | 204.21 | | 10.26 | 184.11  –  224.31 | 19.91 | **3.281e-88** | -91.45 | 9.63 | -110.33  –  -72.58 | -9.50 | **2.187e-21** | 206.95 | 10.22 | 186.92  –  226.97 | 20.26 | **3.088e-91** |
| Block | -37.10 | | 6.50 | -49.84  –  -24.36 | -5.71 | **1.152e-08** | 36.26 | 6.15 | 24.20  –  48.32 | 5.89 | **3.805e-09** | -17.54 | 5.01 | -27.36  –   -7.72 | -3.50 | **4.622e-04** |
| Confidence | -16.10 | | 13.12 | -41.81  –  9.60 | -1.23 | 2.195e-01 | 18.97 | 10.66 | -1.93  –  39.86 | 1.78 | 7.521e-02 | -71.20 | 8.02 | -86.91  –  -55.49 | -8.88 | **6.510e-19** |
| Block : Confidence | 30.09 | | 6.74 | 16.87  –  43.30 | 4.46 | **8.110e-06** | -64.48 | 6.92 | -78.06 –  -50.91 | -9.31 | **1.240e-20** | 16.99 | 6.08 | 5.07  –  28.90 | 2.79 | **5.198e-03** |
| **Random Effects** | | | | | | | | | | | | | | | | |
| Residuals^2^ | | 30497.33 | | | | | 32243.17 | | | | | 26425.43 | | | | |
| Intercept | | 3956.08 | | | | | 3442.90 | | | | | 3972.30 | | | | |
| Confidence | | 5556.10 | | | | | 3218.71 | | | | | 1572.42 | | | | |
| Block | | 1312.50 | | | | | 1113.46 | | | | | 688.02 | | | | |
| N | | 40 | | | | | 40 | | | | | 40 | | | | |
| Observations | | 9996 | | | | | 9996 | | | | | 9996 | | | | |
| Deviance | | 131861.869 | | | | | 132387.369 | | | | | 130376.103 | | | | |
| log-Likelihood | | -65930.935 | | | | | -66193.684 | | | | | -65188.052 | | | | |

*Formula: DV ~ Block* Confidence + (Block + Confidence |participant); DVs are Error Magnitude, RPE and SPE; Note: “:” indicates interactions*
