# Supplementary material for: Response-based outcome predictions and confidence regulate feedback processing and learning
Source: eLife. 2021 Apr 30;10:e62825. doi: 10.7554/eLife.62825 (PMC8121545; doi:10.7554/eLife.62825)
Supplement: Supplementary file 6. [file elife-62825-supp6.docx]

**Table S6.** *Follow-up on* *Block and Confidence effects on Error Signals*

|  | **Error Magnitude** | | | | **RPE** | | | | **SPE** | | | |
| --- | --- | --- | --- | --- | --- | --- | --- | --- | --- | --- | --- | --- |
| *Predictors* | *Estimates* | *SE* | *t* | *p* | *Estimates* | *SE* | *t* | *p* | *Estimates* | *SE* | *t* | *p* |
| (Intercept) | 203.22 | 10.31 | 19.71 | **1.730e-86** | -90.70 | 9.70 | -9.35 | **8.971e-21** | 206.39 | 10.24 | 20.16 | **2.084e-90** |
| Block2-1 | -67.95 | 6.91 | -9.83 | **8.194e-23** | 54.82 | 7.00 | 7.83 | **4.952e-15** | -46.12 | 6.21 | -7.43 | **1.116e-13** |
| Block3-2 | -4.57 | 6.97 | -0.66 | 5.120e-01 | 6.07 | 7.07 | 0.86 | 3.906e-01 | 4.09 | 6.27 | 0.65 | 5.147e-01 |
| Block4-3 | -3.29 | 7.07 | -0.47 | 6.413e-01 | 5.65 | 7.17 | 0.79 | 4.307e-01 | 4.27 | 6.37 | 0.67 | 5.030e-01 |
| Block5-4 | -10.02 | 7.22 | -1.39 | 1.653e-01 | 16.30 | 7.33 | 2.22 | **2.613e-02** | -8.05 | 6.51 | -1.24 | 2.158e-01 |
| Block [1] * Confidence | -53.92 | 15.96 | -3.38 | **7.263e-04** | 93.07 | 14.41 | 6.46 | **1.047e-10** | -104.54 | 11.53 | -9.07 | **1.231e-19** |
| Block [2] * Confidence | -17.32 | 15.45 | -1.12 | 2.622e-01 | 30.90 | 13.80 | 2.24 | **2.518e-02** | -63.90 | 11.02 | -5.80 | **6.591e-09** |
| Block [3] * Confidence | -9.58 | 15.20 | -0.63 | 5.287e-01 | 24.07 | 13.50 | 1.78 | 7.468e-02 | -57.71 | 10.77 | -5.36 | **8.398e-08** |
| Block [4] * Confidence | 2.66 | 15.34 | 0.17 | 8.621e-01 | -19.50 | 13.66 | -1.43 | 1.534e-01 | -60.16 | 10.93 | -5.50 | **3.734e-08** |
| Block [5] * Confidence | 0.81 | 15.79 | 0.05 | 9.592e-01 | -36.91 | 14.15 | -2.61 | **9.110e-03** | -67.73 | 11.35 | -5.97 | **2.422e-09** |
| **Random Effects** | | | | | | | | | | | | |
| Residuals | 30249.30 | | | | 32101.35 | | | | 26295.02 | | | |
| Intercept | 4001.20 | | | | 3497.82 | | | | 3989.42 | | | |
| Confidence | 5509.85 | | | | 3396.66 | | | | 1545.22 | | | |
| Block | 1260.21 | | | | 1077.63 | | | | 650.86 | | | |
| N | 40 | | | | 40 | | | | 40 | | | |
| Observations | 9996 | | | | 9996 | | | | 9996 | | | |
| Deviance | 131780.444 | | | | 132344.613 | | | | 130325.232 | | | |
| log-Likelihood | -65890.222 | | | | -66172.306 | | | | -65162.616 | | | |

*Formula: DV ~ Block/Confidence + (Block + Confidence |participant); DVs are Error Magnitude, RPE and SPE; Note: “:” indicates interactions*
