# Supplementary material for: Response-based outcome predictions and confidence regulate feedback processing and learning
Source: eLife. 2021 Apr 30;10:e62825. doi: 10.7554/eLife.62825 (PMC8121545; doi:10.7554/eLife.62825)
Supplement: Supplementary file 7. [file elife-62825-supp7.docx]

**Table S7.** *Block and Confidence effects on Error Signals*

|  | **RPE** | | | | **SPE** | | | |
| --- | --- | --- | --- | --- | --- | --- | --- | --- |
| *Predictors* | *Estimates* | *SE* | *t* | *p* | *Estimates* | *SE* | *t* | *p* |
| (Intercept) | -82.91 | 7.46 | -11.11 | **1.085e-28** | 201.62 | 5.16 | 39.04 | **0.000e+00** |
| Block | 10.30 | 4.81 | 2.14 | **3.228e-02** | 3.65 | 3.34 | 1.09 | 2.736e-01 |
| Confidence | 9.13 | 15.52 | 0.59 | 5.561e-01 | -60.12 | 6.68 | -9.00 | **2.255e-19** |
| Error Magnitude | -728.76 | 7.24 | -100.67 | **0.000e+00** | 572.09 | 7.27 | 78.71 | **0.000e+00** |
| Block : Confidence | -45.59 | 4.94 | -9.23 | **2.641e-20** | 0.10 | 4.76 | 0.02 | 9.839e-01 |
| **Random Effects** | | | | | | | | |
| Residuals | 15947.32 | | | | 16472.17 | | | |
| Intercept | 2080.51 | | | | 934.32 | | | |
| Confidence | 8858.40 | | | | 1160.03 | | | |
| Block | 720.24 | | | | 243.98 | | | |
| N | 40 | | | | 40 | | | |
| Observations | 9996 | | | | 9996 | | | |
| Deviance | 125421.104 | | | | 125603.380 | | | |
| log-Likelihood | -62710.552 | | | | -62801.690 | | | |

*Formula: DV ~ Block* Confidence + Error Magnitude, + (Block + Confidence |participant);*

*DVs are RPE and SPE; Note: “:” indicates interactions*
