# Supplementary material for: Response-based outcome predictions and confidence regulate feedback processing and learning
Source: eLife. 2021 Apr 30;10:e62825. doi: 10.7554/eLife.62825 (PMC8121545; doi:10.7554/eLife.62825)
Supplement: Supplementary file 8. [file elife-62825-supp8.docx]

**Table S8.** *Follow-up analyses on Confidence-weighted Predicted Error Magnitude effects on P3b*

|  | **P3b Amplitude** | | | | |
| --- | --- | --- | --- | --- | --- |
| *Predictors* | *Estimates* | *SE* | *CI* | *t* | *p* |
| (Intercept) | 4.27 | 0.30 | 3.68 – 4.85 | 14.28 | **2.949e-46** |
| Block2-1 | -0.23 | 0.23 | -0.69 – 0.22 | -1.01 | 3.146e-01 |
| Block3-2 | -0.10 | 0.23 | -0.56 – 0.35 | -0.43 | 6.652e-01 |
| Block4-3 | -0.31 | 0.24 | -0.77 – 0.15 | -1.31 | 1.886e-01 |
| Block5-4 | 0.08 | 0.24 | -0.39 – 0.54 | 0.32 | 7.472e-01 |
| Error Magnitude | -1.04 | 0.50 | -2.01 – -0.07 | -2.09 | **3.652e-02** |
| Sensory Prediction Error | 1.50 | 0.40 | 0.71 – 2.29 | 3.73 | **1.944e-04** |
| Block [1] : Confidence | 1.23 | 0.38 | 0.48 – 1.98 | 3.23 | **1.225e-03** |
| Block [2] : Confidence | 0.40 | 0.36 | -0.30 – 1.10 | 1.13 | 2.569e-01 |
| Block [3] : Confidence | 0.02 | 0.36 | -0.67 – 0.72 | 0.07 | 9.473e-01 |
| Block [4] : Confidence | -0.09 | 0.35 | -0.78 – 0.60 | -0.26 | 7.945e-01 |
| Block [5] : Confidence | 0.02 | 0.35 | -0.67 – 0.70 | 0.05 | 9.634e-01 |
| Block [1] : Predicted Error Magnitude | -0.14 | 0.96 | -2.03 – 1.75 | -0.14 | 8.874e-01 |
| Block [2] : Predicted Error Magnitude | 0.52 | 1.00 | -1.43 – 2.47 | 0.52 | 5.999e-01 |
| Block [3] : Predicted Error Magnitude | -0.74 | 1.06 | -2.83 – 1.35 | -0.69 | 4.878e-01 |
| Block [4] : Predicted Error Magnitude | -1.70 | 0.97 | -3.60 – 0.20 | -1.76 | 7.901e-02 |
| Block [5] : Predicted Error Magnitude | -2.13 | 0.82 | -3.74 – -0.52 | -2.59 | **9.493e-03** |
| Block [1] : Confidence : Predicted Error Magnitude | -5.39 | 1.52 | -8.37 – -2.41 | -3.55 | **3.859e-04** |
| Block [2] : Confidence : Predicted Error Magnitude | -1.63 | 1.54 | -4.66 – 1.39 | -1.06 | 2.895e-01 |
| Block [3] : Confidence : Predicted Error Magnitude | -1.60 | 1.67 | -4.86 – 1.67 | -0.96 | 3.381e-01 |
| Block [4] : Confidence : Predicted Error Magnitude | 3.17 | 1.44 | 0.34 – 6.00 | 2.20 | **2.796e-02** |
| Block [5] : Confidence : Predicted Error Magnitude | 0.83 | 1.16 | -1.43 – 3.10 | 0.72 | 4.721e-01 |
| **Random Effects** | | | | | |
| Residuals | 23.93 | | | | |
| Intercept | 3.27 | | | | |
| Error Magnitude | 3.45 | | | | |
| Confidence | 0.74 | | | | |
| N | 40 | | | | |
| Observations | 9678 | | | | |
| Deviance | 58386.274 | | | | |
| log-Likelihood | -29193.137 | | | | |

*Formula: P3b ~ Block/(Confidence*Predicted Error Magnitude+ SPE)+Error Magnitude + (Error Magnitude+Confidence|participant); Note: “:” indicates interactions*
