# Supplementary material for: Response-based outcome predictions and confidence regulate feedback processing and learning
Source: eLife. 2021 Apr 30;10:e62825. doi: 10.7554/eLife.62825 (PMC8121545; doi:10.7554/eLife.62825)
Supplement: Supplementary file 9. [file elife-62825-supp9.docx]

**Table S9.** *Trial to Trial Improvements by Block and Previous Error and modulations by Previous P3b*

|  | **Improvement** | | | | | **Improvement** | | | | |
| --- | --- | --- | --- | --- | --- | --- | --- | --- | --- | --- |
| *Predictors* | *Estimates* | *SE* | *CI* | *t* | *p* | *Estimates* | *SE* | *CI* | *t* | *p* |
| (Intercept) | -164.33 | 9.53 | -183.00 – -145.66 | -17.25 | **1.102e-66** | -163.29 | 9.78 | -182.46 – -144.12 | -16.69 | **1.491e-62** |
| Block | -0.90 | 3.75 | -8.25 – 6.44 | -0.24 | 8.094e-01 | -3.27 | 4.88 | -12.83 – 6.28 | -0.67 | 5.019e-01 |
| Error Magnitude (n-1) | 0.85 | 0.01 | 0.83 – 0.87 | 84.55 | **0.000e+00** | 0.84 | 0.01 | 0.81 – 0.86 | 68.75 | **0.000e+00** |
| Block : Error Magnitude (n-1) | 0.13 | 0.01 | 0.11 – 0.15 | 10.47 | **1.159e-25** | 0.15 | 0.02 | 0.12 – 0.18 | 9.84 | **7.849e-23** |
| P3b (n-1) |  |  |  |  |  | -0.87 | 3.51 | -7.75 – 6.00 | -0.25 | 8.036e-01 |
| P3b (n-1) : Error Magnitude (n-1) |  |  |  |  |  | 0.01 | 0.01 | -0.01 – 0.03 | 0.88 | 3.765e-01 |
| Block : P3b (n-1) |  |  |  |  |  | 4.34 | 4.86 | -5.18 – 13.86 | 0.89 | 3.715e-01 |
| Block : P3b (n-1) : Error Magnitude (n-1) |  |  |  |  |  | -0.03 | 0.01 | -0.06 – -0.00 | -2.16 | **3.058e-02** |
| **Random Effects** | | | | | | | | | | |
| Residual | 37789.84 | | | | | 36777.96 | | | | |
| Intercept | 3327.45 | | | | | 3324.68 | | | | |
| N | 40 | | | | | 40 | | | | |
| Observations | 9956 | | | | | 9638 | | | | |
| Deviance | 133313.382 | | | | | 128797.375 | | | | |
| log-Likelihood | -66656.691 | | | | | -64398.688 | | | | |

*Formula: Improvement ~ Block* Previous Error Magnitude + (1|participant); Improvement ~ Block* Previous Error Magnitude*Previous P3b + (1|participant)*

*Note: “:” indicates interactions*
