# Supplementary material for: Response-based outcome predictions and confidence regulate feedback processing and learning
Source: eLife. 2021 Apr 30;10:e62825. doi: 10.7554/eLife.62825 (PMC8121545; doi:10.7554/eLife.62825)
Supplement: Supplementary file 10. [file elife-62825-supp10.docx]

**Table S10.** *Follow-up on* *Trial to Trial Improvements by Block and Previous Error and modulations by Previous P3b*

|  | **Improvement** | | | | | **Improvement** | | | | |
| --- | --- | --- | --- | --- | --- | --- | --- | --- | --- | --- |
| *Predictors* | *Estimates* | *SE* | *CI* | *t* | *p* | *Estimates* | *SE* | *CI* | *t* | *p* |
| (Intercept) | -167.22 | 9.69 | -186.21 – -148.23 | -17.26 | **9.611e-67** | -166.61 | 9.91 | -186.04 – -147.18 | -16.81 | **2.155e-63** |
| fblock2-1 | -17.90 | 8.37 | -34.30 – -1.49 | -2.14 | **3.251e-02** | -27.65 | 11.14 | -49.48 – -5.83 | -2.48 | **1.303e-02** |
| fblock3-2 | 10.61 | 8.54 | -6.13 – 27.34 | 1.24 | 2.141e-01 | 22.74 | 11.17 | 0.84 – 44.64 | 2.04 | **4.183e-02** |
| fblock4-3 | 1.24 | 8.53 | -15.48 – 17.97 | 0.15 | 8.841e-01 | -8.19 | 11.00 | -29.75 – 13.38 | -0.74 | 4.568e-01 |
| fblock5-4 | 11.99 | 8.43 | -4.54 – 28.52 | 1.42 | 1.552e-01 | 15.11 | 10.68 | -5.83 – 36.05 | 1.41 | 1.572e-01 |
| Block[1] * EM (n-1) | 0.66 | 0.02 | 0.63 – 0.69 | 43.37 | **0.000e+00** | 0.62 | 0.02 | 0.59 – 0.66 | 33.62 | **7.714e-248** |
| Block[2] * EM (n-1) | 0.94 | 0.02 | 0.90 – 0.99 | 43.44 | **0.000e+00** | 0.96 | 0.03 | 0.91 – 1.01 | 36.48 | **2.119e-291** |
| Block[3] * EM (n-1) | 0.92 | 0.02 | 0.87 – 0.96 | 38.53 | **0.000e+00** | 0.89 | 0.03 | 0.84 – 0.95 | 29.77 | **9.296e-195** |
| Block[4] * EM (n-1) | 0.91 | 0.02 | 0.86 – 0.95 | 40.68 | **0.000e+00** | 0.91 | 0.03 | 0.86 – 0.97 | 32.33 | **2.862e-229** |
| Block[5] * EM (n-1) | 0.90 | 0.02 | 0.85 – 0.95 | 37.24 | **1.711e-303** | 0.90 | 0.03 | 0.85 – 0.96 | 31.28 | **7.981e-215** |
| Block[1] * P3b (n-1) |  |  |  |  |  | -8.39 | 7.85 | -23.78 – 7.00 | -1.07 | 2.854e-01 |
| Block[2] * P3b (n-1) |  |  |  |  |  | 11.45 | 7.72 | -3.69 – 26.58 | 1.48 | 1.382e-01 |
| Block[3] * P3b (n-1) |  |  |  |  |  | -8.10 | 7.92 | -23.62 – 7.42 | -1.02 | 3.063e-01 |
| Block[4] * P3b (n-1) |  |  |  |  |  | 4.19 | 7.71 | -10.92 – 19.31 | 0.54 | 5.865e-01 |
| Block[5] * P3b (n-1) |  |  |  |  |  | 0.81 | 7.59 | -14.07 – 15.69 | 0.11 | 9.151e-01 |
| Block[1] * P3b (n-1) * EM (n-1) |  |  |  |  |  | 0.06 | 0.02 | 0.03 – 0.10 | 3.35 | **8.209e-04** |
| Block[2] * P3b (n-1) * EM (n-1) |  |  |  |  |  | -0.04 | 0.03 | -0.09 – 0.01 | -1.48 | 1.389e-01 |
| Block[3] * P3b (n-1) * EM (n-1) |  |  |  |  |  | 0.02 | 0.03 | -0.04 – 0.08 | 0.71 | 4.805e-01 |
| Block[4] * P3b (n-1) * EM (n-1) |  |  |  |  |  | -0.01 | 0.03 | -0.06 – 0.04 | -0.38 | 7.030e-01 |
| Block[5] * P3b (n-1) * EM (n-1) |  |  |  |  |  | -0.01 | 0.03 | -0.07 – 0.05 | -0.31 | 7.566e-01 |
| **Random Effects** | | | | | | | | | | |
| Residual | 37335.51 | | | | | 36296.90 | | | | |
| Intercept | 3453.25 | | | | | 3427.28 | | | | |
| N | 40 | | | | | 40 | | | | |
| Observations | 9956 | | | | | 9638 | | | | |
| Deviance | 133194.844 | | | | | 128672.144 | | | | |
| log-Likelihood | -66597.422 | | | | | -64336.072 | | | | |

*Formula: Improvement ~ Block/Previous Error Magnitude + (1|participant); Improvement ~ Block/(Previous Error Magnitude*Previous P3b) + (1|participant)*

*Note: “:” indicates interactions*
